# Shape memory materials based on adamantane-containing polyurethanes†

**DOI:** 10.1039/c8ra05111a

**Published:** 2018-07-18

**Authors:** Faxing Zou, Heng Chen, Shuqin Fu, Shaojun Chen

**Affiliations:** Guangdong Research Center for Interfacial Engineering of Functional Materials, Shenzhen Key Laboratory of Special Functional Materials, College of Materials Science and Engineering, Shenzhen University Shenzhen 518060 China polyhengchen@szu.edu.cn chensj@szu.edu.cn

## Abstract

In this contribution, a series of adamantane (AD)-containing polyurethanes were prepared from 1,3-adamantanediol, 1,4-butanediol and hexamethylene diisocyanate, and the influences of AD on the shape memory behavior of polyurethanes were systematically studied. Due to large steric hindrance, AD was able to disrupt the regular arrangement of polyurethane chains and contributed to forming an amorphous domain. It was found that moderate AD-containing polyurethanes had good mechanical properties and broad glass transition, and shape memory tests confirmed these polyurethanes possessed a shape fixation rate of 98% and shape recovery rate of 91% during a dual-shape memory procedure. Furthermore, they also exhibited a triple-shape memory effect. This work demonstrated a facile and feasible way to prepare polyurethane-based shape memory materials by using adamantane as a functional unit.

## Abstract 2

A series of adamantane (AD)-containing polyurethanes were prepared from 1,3-adamantanediol, 1,4-butanediol and hexamethylene diisocyanate, and the influences of AD on the shape memory behavior of polyurethanes were systematically studied.

## Graphical abstract

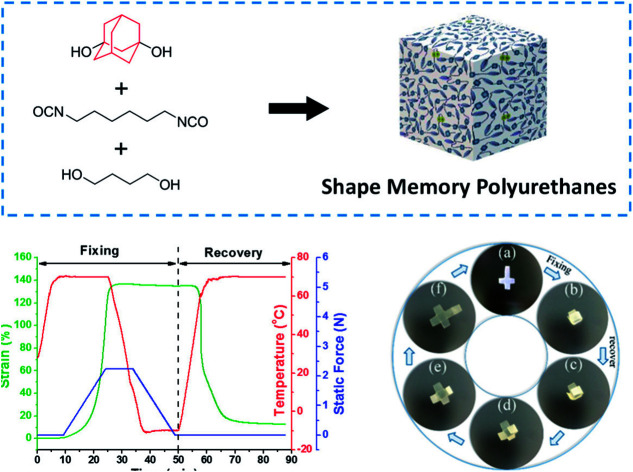

## 1. Introduction

Shape memory polymers (SMPs) are a class of smart materials which are able to “memorize” a permanent shape. SMPs can be fixed to one temporary shape under appropriate conditions, and also recover to their permanent shape in response to an external trigger, *e.g.* heat, light, or water, or magnetism.^1^ Compared with their alloy counterparts, shape memory alloys, SMPs exhibited many advantages, including low density, extremely high recoverable strain, broad shape recovery temperature range, a variety of different stimulating methods, *etc.*^2^ The intelligent technology has given rise to many interesting developments in both industry as well as academia, and now it has been applied in many fields, such as shrinkable tubes,^3^ smart fabrics,^4^ solar sails of spacecraft,^5^ and surgery implants.^6,7^

In general, SMPs possess a dual segment/domain structure where one “hard” segment/domain is always elastic to maintain permanent shape and the other “soft” one is transitionable in the presence of an appropriate stimulus.^8^ Up till now, various polymer systems have been exploited for shape memory behaviour, including polyethylenes,^9^ polystyrenes,^10^ polyurethanes,^11^ polyacrylates,^12^ and *etc.*^13,14^ Among them, polyurethanes receive distinctive attentions for facile preparation, good biocompatibility, and especially tunable properties. Polyurethanes are normally made of tangled long linear chains consisting of two types of alternatively connected segments where the structural units derived from diisocyanate and chain extenders constitute the hard segments whereas those from diisocyanate and macroglycols convert to the soft segments. By introducing functional units, the soft segments are well designed and versatile performances of SMPs will be obtained. Jing *et al.* used poly(ε-caprolactone) as soft segments of polyurethanes, and lowest recovery temperature of SMPs was controlled in the range of 37–42 °C, and reasonable rigidity could be retained after shape recovery, fulfilling the essential requirements of medical implantations.^15^ Hu *et al.*, found polyurethanes containing pyridine moieties had excellent moisture absorption properties, which was based on the dissociation or disrupt of hydrogen bonding in the pyridine-ring induced by moisture absorption.^16^ Luo *et al.*, found introduction of zwitterionic units into polyurethanes significantly improved hydrophilicity and reduced protein adsorption.^17^ Later, Chen *et al.* demonstrated zwitterionic polyurethanes had multi-shape memory effects, moisture-sensitive shape memory effect, and self-healing properties.^18,19^ These works greatly promoted the development of polyurethane-based SMPs, and further work should be carried on it.

Adamantane (AD) is a symmetric and three-dimensional hydrocarbon, and it exhibits remarkable structural, physical and chemical properties, such as high rigidity, polarizability, hydrophobicity, and promising medicinal activity.^20^ Insertion of AD into the backbones of polymers, including polysulfones,^21^ polyesters,^22^ polyamides,^23,24^ and polyimides,^25,26^ has been found to enhance thermal stability and glass-transition temperature, increase chain stiffness, decrease crystallinity, and lower dielectric constant values. These property improvements should also be applicable to polyurethanes and have promising influences on its shape memory effect.

To our best knowledge, the influence of AD on the microstructure and shape memory effect of polyurethanes has never studied. Inspired by it, a series of polyurethanes with AD in their backbones were prepared from 1,3-adamantanediol/1,4-butanediol/hexamethylene diisocyanate, and their structure, thermal properties, mechanical properties, and shape memory behaviours were systematically investigated.

## 2. Experimental section

### 2.1 Materials

1,3-Adamantanediol (ADO, 99%), hexamethylene diisocyanate (HDI, 99%), 1,4-butanediol (BDO, 99%), dibutyltin dilaurate (DBTDL, 95%), and *N*,*N*-dimethylformamide (DMF, 99.5%) were purchased from Aladdin Chemical Reagent Co. Ltd (Shanghai, China) and were used without further purification.

### 2.2 Synthesis of AD-containing polyurethanes

**Scheme 1:**
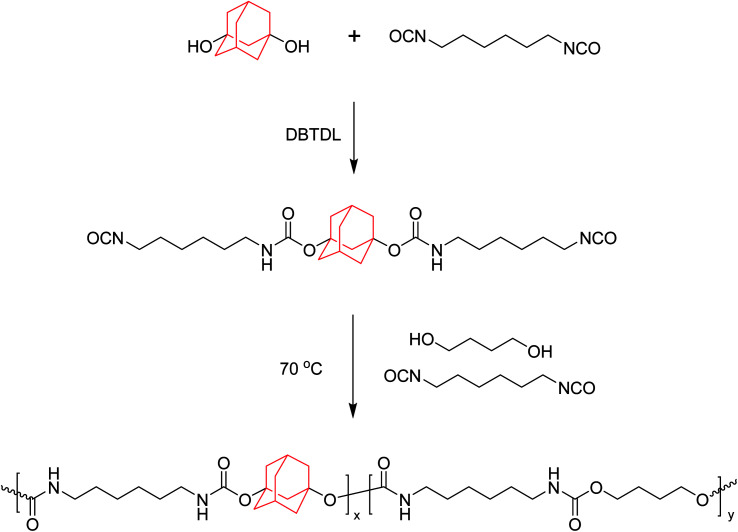
Synthesis route of ABPUs.

The synthetic route of AD-containing polyurethanes is shown in Scheme 1. The synthesis of polyurethanes was carried out in a 100 mL flask filled with nitrogen and equipped with a mechanical stirrer, a thermometer, and a condenser. Typically, ADO, HDI, DBTDL, and 10 mL DMF were mixed in the flask, and the mixture was stirred vigorously at 70 °C for 30 min. Then BDO was added to the flask, followed by stirring at 70 °C for 2 h. Meanwhile, 10 mL of DMF was occasionally added to the reaction to reduce the viscosity of the solution. After the reaction was completed, the resulting solution was poured into a Teflon pan and DMF was removed by evaporation at 80 °C for 24 h in air flow then, dried in vacuum for another 24 h. The detailed feed ratios are listed in Table 1, and samples are denoted as ABPU*x*, where *x* refers to the ADO/HDI molar ratio multiplied by 100.

**Table 1:** The composition of ABPUs

Samples	ADO (mmol)	BDO (mmol)	HDI (mmol)	*M*_n_ a	PDI^b^
ABPU00	0	10	10	34 200	1.81
ABPU20	2	8	10	53 700	2.12
ABPU40	4	6	10	66 700	1.96
ABPU50	5	5	10	61 900	2.36
ABPU60	6	4	10	52 900	2.07
ABPU80	8	2	10	58 800	2.42
ABPU100	10	0	10	42 800	2.15

a *M*_n_ is the number-average molecular weight.

b PDI is the polydispersity index.

### 2.3 Characterization

Nuclear magnetic resonance (NMR) measurements were recorded using a Bruker AV400 spectrometer (Bruker BioSpin, Switzerland) with tetramethylsilane (TMS) as an internal standard and CDCl_3_ DMSO-*d*_6_ as solvent. Fourier transform infrared spectra (FTIR) were recorded using a Nicolet 760 IR spectrometer, and each spectrum was collected over 32 scans with a spectral resolution of 4 cm^−1^. Gel permeation chromatography (GPC) measurement was conducted on a Waters E2695 Alliance system (Waters, USA) with DMF as the eluent and polystyrene as the internal standard. TGA experiment was carried out on a TA Instruments (TGA Q50) system with the scanning range from 100 to 600 °C and a heating rate of 10 °C min^−1^ in a N_2_ flow (60 mL min^−1^). Differential scanning calorimetry (DSC) analysis was performed using a TA Q200 instrument with a heating/cooling rate of 10 °C min^−1^. Dynamic mechanical analysis (DMA) was performed with TA universal Q800 (TA, USA) and specimens were determined under 1.0 Hz and a heating rate of 2.0 K min^−1^. Atomic force microscopy (AFM) was conducted using Dimension Icon (Bruker, USA) in tapping mode (resonance frequency ∼300 kHz, spring constant 40 N m^−1^). The samples were dissolved in DMF at a concentration of 5 mg mL^−1^ and spin-coated at 400 rpm for 10 s and then, at 4000 rpm for 60 s on oxidized silicon substrates. Spin-coated films were kept in an oven at 50 °C for 48 h to evaporate the solvent.

### 2.4 Testing of shape-memory behaviours

Shape memory behaviours were evaluated *via* thermo-mechanical analysis using a TA Instruments (DMA800) and using tension clamps in controlled force mode, according to a reported procedure with some modifications.^27^ All samples were dried at 80 °C in vacuum for 24 h and cut in rectangular pieces of approximately 15 mm × 4.0 mm × 0.5 mm. The detailed test setup of dual- and triple-shape-memory cycles are described as follows. All the measurements of ABPUs were repeated three times for each specimen.

#### Dual-shape memory cycle

(1) The sample was heated at *ca. T*_g_ + 20 °C and equilibrated for 10 min; (2) uniaxial stretching was applied to reach a predetermined strain (*ε*_load_) by ramping the force from 0.001 to 1 N with a rate of 0.1 N min^−1^, after that, the sample was allowed to equilibrate for 1 min; (3) the strain was fixed by rapid cooling to *ca.* 0 °C with a cooling rate of 10 °C min^−1^, equilibrated for 10 min; (4) the external force was unloaded to 0 N with a rate of 0.15 N min^−1^, and the strain was recorded as *ε*_X_; (5) the sample was reheated to *ca. T*_g_ + 20 °C with a rate of 10 °C min^−1^ and followed by equilibration for 40 min. Finally, the remained strain was recorded as *ε*_rec_. The shape fixation (*R*_f_) and shape recovery (*R*_r_) of dual-shape memory were calculated according to the following equations.^28^1
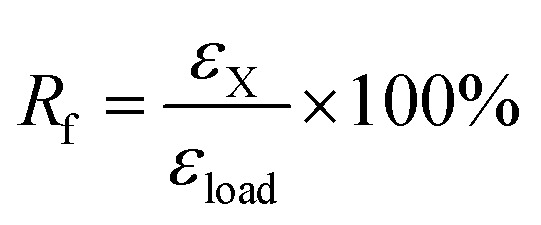
2
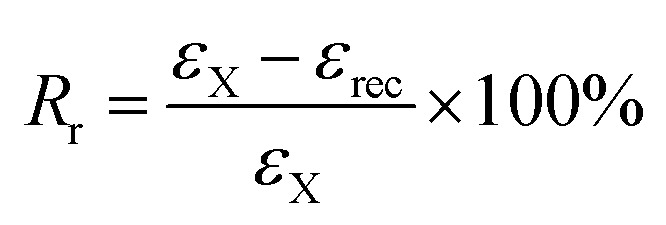


#### Triple-shape memory cycles

(1) The sample was heated to 75 °C and equilibrated for 5 min; (2) uniaxial stretching was applied to reach a predetermined strain (*ε*_X,load_) by ramping the force from 0.001 N to 5 N at a rate of 0.15 N min^−1^, and then the sample was equilibrated for 3 min; (3) the strain was fixed by rapid cooling to 55 °C at a cooling rate of 10 °C min^−1^, followed by equilibration for 10 min. Then external force was unloaded to 0 N at a rate of 0.15 N min^−1^, and the strain was recorded as *ε*_X_; (4) uniaxial stretching was applied to reach another predetermined strain as *ε*_Y,load_ by ramping the force from 0.001 N to 10 N at a rate of 0.15 N min^−1^, and then the sample was equilibrated for 3 min; (5) the strain was further fixed by rapid cooling to 0 °C with a cooling rate of 10 °C min^−1^, followed by equilibration for 10 min. Then external force was unloaded to 0 N at a rate of 0.15 N min^−1^, and the strain was recorded as *ε*_Y_; (6) the sample was reheated to 55 °C at a rate of 10°C min^−1^ and followed by equilibration for 30 min, and the strain was recorded as *ε*_X,rec_; (7) finally, the sample was reheated to 75 °C at a rate of 10 °C min^−1^ and followed by equilibration for 40 min. The shape fixation (*R*_f_) and shape recovery (*R*_r_) of triple-shape memory effect were calculated according to the following equations,^27^3
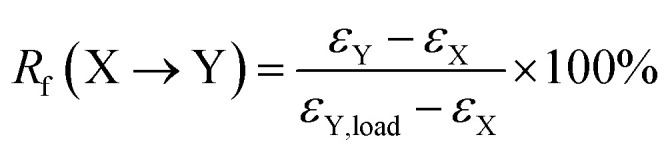
4
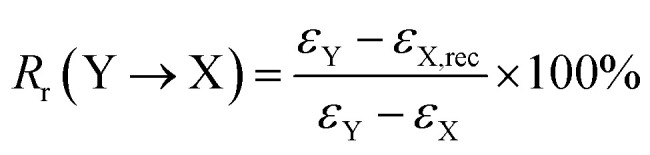
where X and Y denote two different temporary shapes, respectively.

## 3. Results and discussions

### 3.1 Synthesis and characterization of ABPUs

**Fig. 1:**
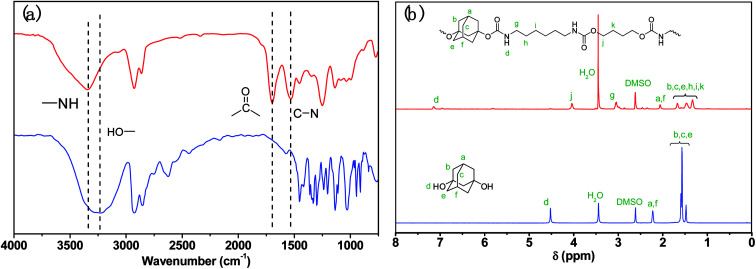
(a) FTIR spectra of ADO (blue curve) and ABPU (ABPU50, red curve); (b) ^1^H NMR spectra of ADO (blue curve) and ABPU (ABPU50, red curve).

In this study, the ABPUs were synthesized by the polyaddition of ADO, BDO and HDI under the catalysis of DBTDL. The structure of APBUs was characterized by FTIR, ^1^H NMR and GPC. The FTIR spectra are shown in Fig. 1a. Compared to reactant of ADO, the characteristic peak of –OH at 3250 cm^−1^ disappeared in the spectrum of ABPU, and instead new peaks appeared at 1530, 1690, and 3350 cm^−1^, corresponding to C–N, –C=O, and –NH bonds, respectively.^29^ In the ^1^H NMR spectra (Fig. 1b), the signal of –OH on ADO disappeared, and that of –NH on ABPU was observed at 7.15 ppm,^30^ meanwhile, the signals located at 1.50, 3.05, and 4.05 ppm were attributed to protons of AD, decoyl, and butyl, respectively.^31–33^ Furthermore, molecular weight of ABPUs was determined by GPC, and the *M*_n_ ranges from 34 200 to 66 700. These results confirm that AD-containing polyurethanes were successfully synthesized.

### 3.2 Thermal properties of ABPUs

**Fig. 2:**
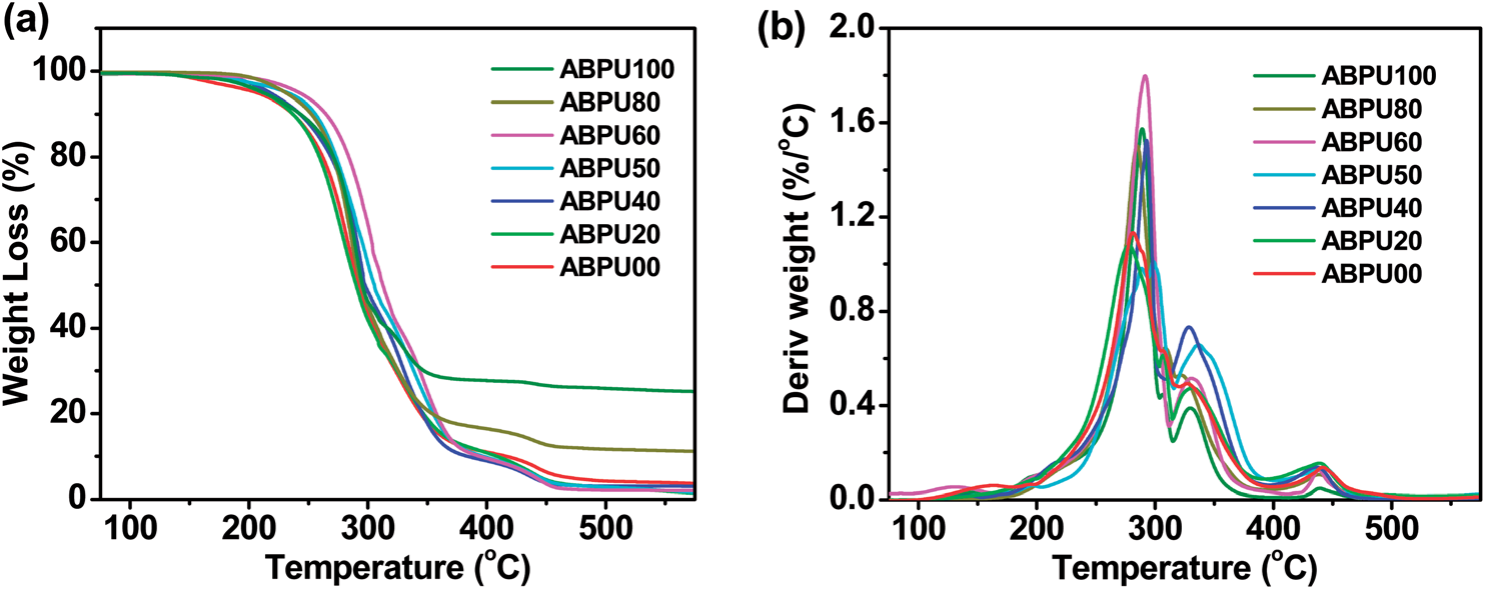
TGA (a) and DTG (b) curves of ABPUs.

**Fig. 3:**
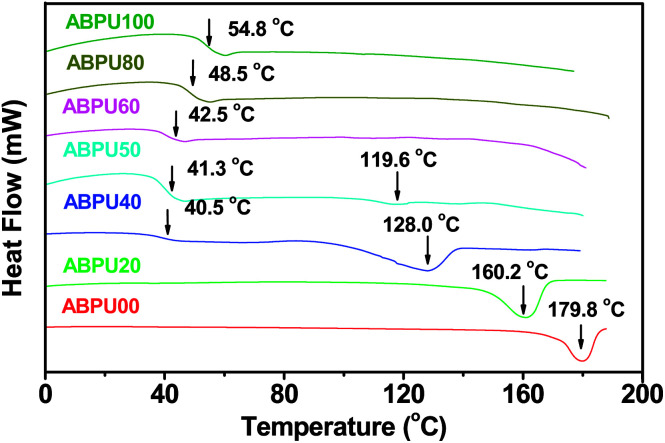
DSC curves of ABPUs.

Thermal properties of ABPUs were investigated using TGA and DSC. Fig. 2 presents the TGA and DTG curves of ABPUs. All ABPUs exhibited degradation temperature at about 240 °C, and this indicates that these polyurethanes possess good thermal stability. In the DSC curves (Fig. 3), low AD-containing samples, typically as ABPU00, showed an endothermic peak in the temperature region of 100–200 °C, and the peak corresponds to the melting temperature of crystalline phase, and this indicated these low AD-containing polyurethanes were crystalline. Meanwhile, high AD-containing samples exhibited obvious glass transition between 40 and 60 °C, suggesting these high AD-containing polyurethanes were amorphous. As the content of AD increased, the melting peak shifted towards low temperature and became weak. These changes were due to the introduced AD units that disrupted regular arrangement of polyurethanes chains and contributed to the conversion form crystalline form to amorphous form. Furthermore, the glass transition shifted towards high temperature with the increasing content of AD, and this was because that AD segments has poor mobility for its large steric hindrance.

### 3.3 Mechanical properties

**Fig. 4:**
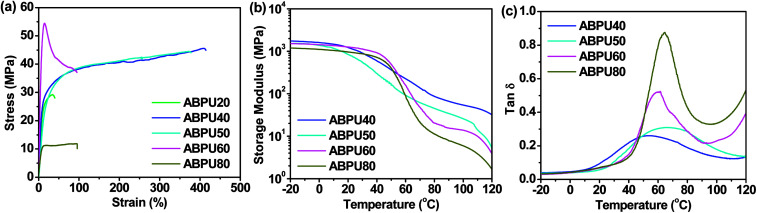
(a) Tensile stress–strain curves of ABPUs; (b) DMA storage modulus curves of ABPUs; (c) loss factor (tan *δ*) curves of ABPUs.

Mechanical properties are crucial to function implementation of shape memory materials, herein static and dynamic mechanical properties of ABPUs were investigated by tensile tests and DMA, respectively. The samples of ABPUs were prepared by solvent casting method, however, the samples of ABPU00 and ABPU100 were failed to form film. This was mainly because that polyurethane precipitated, for crystallization or large steric hindrance of AD, before film formation.^34^ The stress–strain curves of ABPUs are shown in Fig. 4a and detailed result are listed in Table S1.† Only ABPU 40 and ABPU50 exhibited ductile fractures. Their breakage elongations are larger than 370% and tensile strength are higher than 44 MPa. This result suggested moderate introduction of AD contributed to enhance flexibility of polyurethanes and maintain its high strength. The high flexibility and strength of moderate AD-containing polyurethanes will be beneficial to the implementation of their shape memory application. Dynamic mechanical properties of ABPUs were investigated using DMA, and a significant temperature dependence of dynamic mechanical properties was observed. Fig. 4b and c shows the storage modulus (*E*′) and tan *δ* for ABPUs. The storage modulus of ABPUs showed a plateau regime below 40 °C, and it underwent a sharp decrease as the temperature increased, indicating a glass transition. The detailed relaxation was studied based on the tan *δ* curves of ABPUs (Fig. 4c). All ABPUs exhibited a single peak below 110 °C, which corresponded to the glass transition. Compared to high AD-containing samples, moderate AD-containing samples showed a broader peak, and this broad transition can be viewed as the collective contribution of an infinite number of transitions.^35^ Due to large steric hindrance of AD, it hampers the formation of hydrogen bonding of urethane groups. In the dense zone of AD, the hydrogen bonding of urethane groups is weak and tends to cause a low temperature transition, while in its sparse zone, the hydrogen bonding of urethane groups is strong and results to a high temperature transition. Thus, the uneven distribution of AD caused a series of sharp glass transitions, and they continuously distributed to form the broad transition. The low-temperature and high-temperature transition corresponds to soft and hard segment of shape memory materials, and the broad glass transition will contribute to multi-shape memory effect of these polyurethanes.

### 3.4 Morphology analysis

**Fig. 5:**
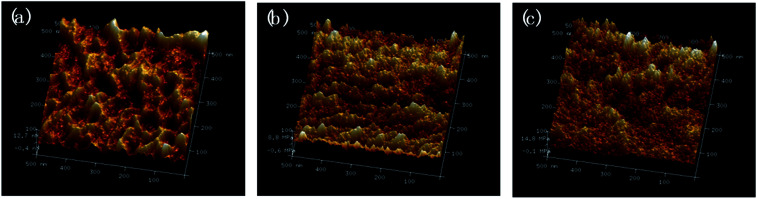
AFM 3D DMT modulus images of ABPU40 (a), ABPU50 (b), and ABPU60 (c).

**Fig. 6:**
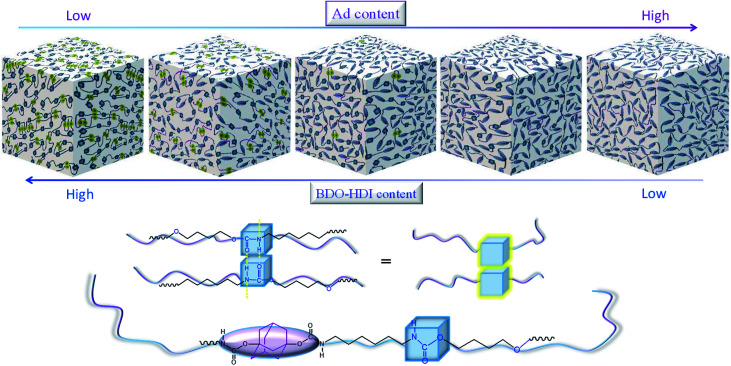
Illustration of structure change of ABPUs.

The morphology of ABPUs were further investigated using AFM with the Derjaguin–Mueller–Toporov (DMT) model where a region of elevated light indicates high Young's modulus.^36^ AFM 3D DMT images of ABPUs, shown in Fig. 5, exhibited an obviously uneven surface suggested a phase separation structure. Some cuspidal outshoots appear throughout the surface which might be the aggregation of hard segments, while depression region should be that of soft segments. As the content of AD increased, cuspidal outshoots reduced, and this suggested hard phase was disrupted by AD. This result confirmed that AD disturbed the microstructure of polyurethanes and contributed to form a soft phase. This phenomenon can be elaborated as illustrated in Fig. 6. The segments derived from BDO and HDI have high intramolecular interactions, especially the hydrogen bonds between urethane–urethane, due to its high dense urethane groups, and they are prone to form crystalline structures. As AD was introduced, its large steric hindrance weakened these high intramolecular interactions and forced polyurethane chains to form amorphous domains. These amorphous domains with high dense of AD have low intramolecular interactions would constitute new soft phase of polyurethanes. The weakening trend of hydrogen bond was demonstrated by FTIR analysis where characteristic peak of –NH bonds shifted to lower wavenumbers with the increasing of AD (Fig. S1†).

### 3.5 Thermal-induced multi-shape memory effects

**Fig. 7:**
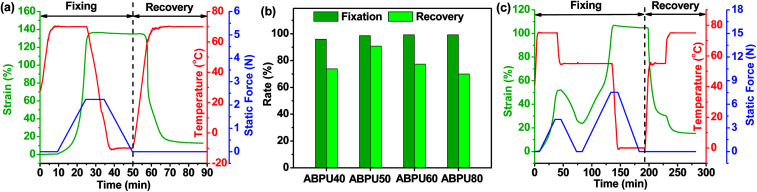
(a) Dual-shape memory behaviors of ABPU50. (b) Shape fixation and recovery rates of ABPUs. (c) Triple-shape memory behavior of ABPU50.

**Fig. 8:**
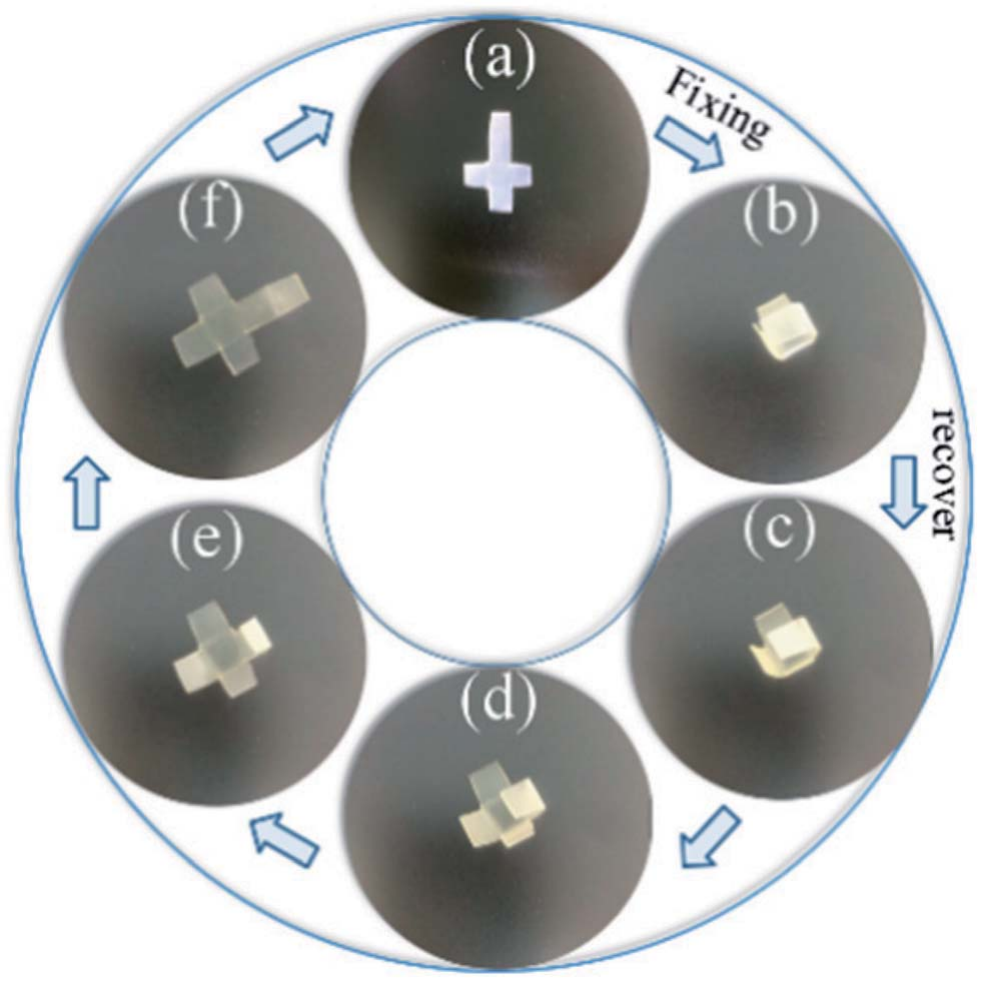
Illustration of ABPU50 in self-unpackaging.

As analyzed above, dynamic mechanical properties of ABPUs exhibited significant temperature dependence, and this allowed ABPUs to be used as thermal-induced shape memory materials. The thermal-induced SMEs of ABPUs were investigated by thermo-mechanical analysis using a TA Instruments DMA. In the dual-shape memory procedure, the sample was deformed by stretching at *T*_g_ + 20 °C, fixed at 0 °C, and recovered at *T*_g_ + 20 °C. These procedures are illustrated in Fig. 7a, and the shape fixation (*R*_f_) and shape recovery rate (*R*_r_) are listed in Fig. 7b. All ABPUs samples exhibited a good *R*_f_ value higher than 95%, however, the *R*_r_ value were relative low, and only that of ABPU50 reached above 90%. The *R*_r_ of polyurethanes has been confirmed to be mainly related to the content of hard segment.^37,38^ The shape recovery procedures of ABPUs were carried out at *T*_g_ + 20 °C, and this temperature can be regarded as a cut-off point between hard segments and soft segments. Fig. 3b revealed ABPU50 exhibited a broad glass transition, and this indicated it also possessed high content of hard segments. Therefore, *R*_r_ value of ABPU50 was relative higher. Furthermore, the multi-shape memory recovery of ABPU50 was also investigated. The triple-shape memory procedure is shown in Fig. 7c. The ABPU50 sample was stretched to about 50% strain at 75 °C, fixed at 55 °C, stretched to another 50% strain, and fixed at 0 °C, sequentially. Upon heating, it was found that the shape of ABPU50 was recovered from the 100% strain to 30% strain at 55 °C, and to *ca.* 15% strain at 75 °C. The *R*_f_ (X → Y) and *R*_r_ (Y → X) of its triple-shape memory were *ca.* 97.6% and *ca.* 92.5%, suggesting a good multi-shape memory effect. As analyzed above, the broad transition of ABPU50 can be regarded as many individual memory elements, during the triple memory procedure, two operating temperatures of 75 and 55 °C activated two different memory elements of soft segments, leading to the triple memory effect. Given the multi-shape memory effect, the application of ABPU50 in self-unpackaging was attempted (Fig. 8). A cross-type film of ABPU50 was crafted into a cubic box at 80 °C, and the temporary shape was fixed by cooling to 0 °C. Upon heating, the cubic box would be opened automatically in a minute (see ESI†). These demonstrations confirmed that the adamantane-containing polyurethanes can be used as shape memory materials for many applications, such as self-unpacking devices, actively moving equipment, and intelligent medical implants.

## 4. Conclusions

In summary, shape memory materials based on adamantane-containing polyurethanes were developed by facilely introducing 1,3-adamantanediol to 1,4-butanediol/hexamethylene diisocyanate system. Through scrutinizing a series of polyurethanes with different feed ratio, it was found that moderate AD-containing polyurethanes showed good mechanical properties. More importantly, the moderate AD-containing polyurethanes exhibited a broad glass transition, which was probably due to the uneven distribution of AD that had large steric hindrance and weakened hydrogen bonding among urethane groups. These good properties made moderate AD-containing polyurethanes available for shape memory materials. The moderate AD-containing polyurethanes exhibited a shape fixation rate of 98% and shape recovery rate of 91% during dual-shape memory procedure, and it also exhibited a triple-shape memory effect. And, further study of AD-containing polyurethanes on biological applications is ongoing.

## Conflicts of interest

There are no conflicts to declare.

## Supplementary Material

RA-008-C8RA05111A-s001

RA-008-C8RA05111A-s002
