# Supplementary material for: Shape memory materials based on adamantane-containing polyurethanes
Source: RSC Adv. 2018 Jul 18;8(45):25584–91. doi: 10.1039/c8ra05111a (PMC9082574; doi:10.1039/c8ra05111a)
Supplement: RA-008-C8RA05111A-s002 [file RA-008-C8RA05111A-s002.pdf]

Electronic Supplementary Material (ESI) for RSC Advances.
This journal is © The Royal Society of Chemistry 2018

Supporting Information

Shape Memory Materials based on Adamantane-containing Polyurethanes

Faxing Zou, Heng Chen*, Shuqin Fu, Shaojun Chen*

Shenzhen Key Laboratory of Functional Polymer, College of Chemistry and Environmental Engineering, Shenzhen University, Shenzhen, 518060, China.
Guangdong Research Center for Interfacial Engineering of Functional Materials; Shenzhen Key Laboratory of Special Functional Materials, College of Materials Science and Engineering, Shenzhen University, Shenzhen 518060, China.

E-mail: polyhengchen@szu.edu.cn (for H. Chen); chensj@szu.edu.cn (for S. Chen);

Keywords: shape memory polymers; adamantane; polyurethanes

Testing procedure of shape-memory behaviours

Thermal-induced shape memory behaviors were investigated with DMA Q800 apparatus (TA, USA) in tensile mode with a preload force of 0.01 N, an oscillation amplitude of 5 μm (< 0.18%), static stress/dynamic stress amplitude ratio (“force tracking”) of 80%, and an oscillation frequency of 1 Hz. Specimens were cut from the final ABPUs film to feature dimensions of 15 mm × 5 mm × 0.4 mm. After loading each film specimen at room temperature under tensile stress, the temperature was ramped to 70 °C at a rate of 10 °C/min. ABPU was thermally equilibrated at 70 °C and stretched to 80% strain (ε_1), elongated to a force of 1.6 N at a rate of 0.15 N/min (in tension); cooled to 10 °C at a rate of 10 °C· min^{-1}, and then unloaded to 0 N using the same rate as before. The remaining strain was measured as ε_2. The specimen was then reheated to 70 °C at a ramp rate of 10 °C/min, and the final strain was recorded as

recovery strain, ε_3. All the above measurements of ABPUs were repeated three times for each specimen.

Multi-shape memory behaviours were evaluated via thermo-mechanical analysis using a TA Instruments (DMA800) and using tension clamps in controlled force mode, according to the procedure described in a literature [27]. All samples were dried at 80 °C in vacuum for 24 h and cut in rectangular pieces of approximately 15 mm × 4.0 mm × 0.5 mm. The detailed test setup of dual- and triple-shape-memory cycles are described as follows.

Dual-shape memory cycle

The sample was heated at *ca.* 100 °C and equilibrated for 10 min; (2) Uniaxial stretching was applied by ramping the force from 0.001 to 1 N with a rate of 0.1 N/min. The sample was then allowed to equilibrate for 1 min; (3) The strain was then fixed by rapid cooling to *ca.* 0 °C with a cooling rate (q) of -10 °C/min, followed by equilibration for 10 min; (4) The external force was then unloaded to 0 N with a rate of 0.15 N/min; (5) Finally, the sample was reheated to *ca.* 70 °C with a rate of 10 °C/min and followed by equilibration for 40 min.

Triple-shape memory cycles.

The sample was heated to 75 °C and equilibrated for 5 min; (2) Uniaxial stretching was applied by ramping the force from 0.001 N to 5 N at a rate of 0.15 N/min. The sample was then allowed to equilibrate for 3 min; (3) The strain was then fixed by rapid cooling to 55 °C at a cooling rate of -10 °C/min, followed by equilibration for 10 min; (4) The external force was unloaded to 0 N at a rate of 0.15 N/min first, and then uniaxial stretching was applied by ramping the force from 0.001 N to 10 N at same rate; (5) The strain was further fixed by rapid cooling to 0 °C with a cooling rate of -10 °C/min, followed by equilibration for 10 min; (6) The external force was unloaded to 0 N at a rate of 0.15 N/min; (7) The sample was reheated to 55 °C at a rate of 10°C/min and followed by equilibration for 30 min; (8) Finally, the sample was reheated to 75 °C at a rate of 10 °C/min and followed by equilibration for 40 min.

Table S1. Tensile Properties of properties of ABPUs

Samples	Break stress (MPa)	Break strain (%)	Yield stress (MPa)	Yield strain (%)	Tangent modulus (MPa)
ABPU20	28.5	39.6	29.1	33.7	265.8
ABPU40	44.9	414.6	45.6	409.5	342.2
ABPU50	44.0	377.5	44.5	372.6	181.8
ABPU60	37.0	95.0	54.3	13.7	670.8
ABPU80	11.9	94.2	11.2	15.4	152.5

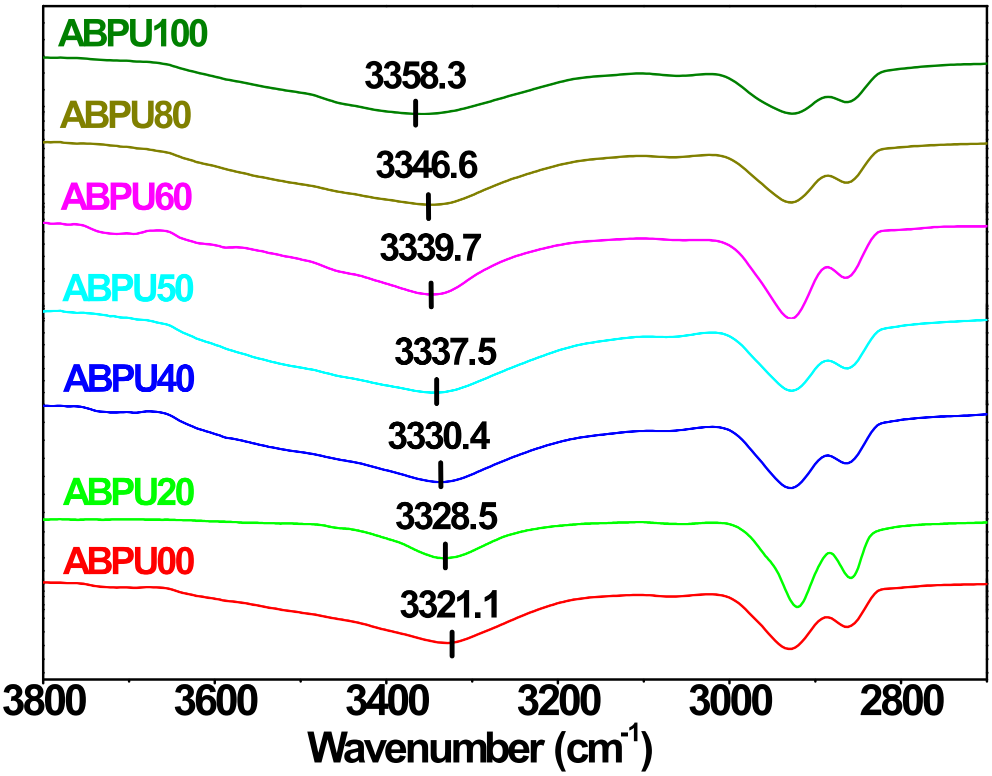

Fig. S1 Partial enlargement FT-IR spectra of ABPUs.
